# Long Noncoding RNA KLF3-AS1 Acts as an Endogenous RNA of miR-223 to Attenuate Gastric Cancer Progression and Chemoresistance

**DOI:** 10.3389/fonc.2021.704339

**Published:** 2021-10-21

**Authors:** Houxiang Jiang, KaiFeng Hu, Yabing Xia, Linhu Liang, Xiaoli Zhu

**Affiliations:** Department of Gastrointestinal Surgery, The First Affiliated Hospital of Wannan Medical College (Yijishan Hospital of Wannan Medical College), Wuhu, China

**Keywords:** gastric cancer, lncRNA, KLF3-AS1, progression, miR-233, chemosensitivity

## Abstract

Gastric cancer is a deadly disease, and the low rate of early diagnosis and chemoresistance largely contributed to the poor prognosis of gastric cancer. LncRNAs have been extensively reported for their roles in regulating cancer progression. In this study, we found that KLF3-AS1 was down-regulated in gastric cancer cells. Overexpression of KLF3-AS1 repressed gastric cancer cell proliferation, growth. In addition, KLF3-AS1 overexpression also exerted inhibitory effects on the gastric cancer cell invasion, migration and EMT, but promoted chemosensitivity of gastric cancer cells to cisplatin. The mechanistic studies showed that KLF3-AS1 could act as the “sponge” for miR-223 and to repress miR-223 expression in gastric cancer cells. Overexpression of miR-223 reversed the inhibitory effects of KLF3-AS1 overexpression on gastric cancer cell proliferation, invasion, migration and EMT, and attenuated the enhanced effects of KLF3-AS1 overexpression on gastric cancer cell chemosensitivity to cisplatin. The *in vivo* studies showed that KLF3-AS1 overexpression suppressed the tumor growth of SGC-7901 in the nude mice. In conclusion, our results for the first time demonstrated that KLF3-AS1 was down-regulated in gastric cancer cells and repressed gastric cancer cell proliferation, invasion, migration and EMT, and enhanced chemosensitivity to cisplatin. Further mechanistic results indicated that KLF3-AS1 exerted its biological function in gastric cancer cells by inhibiting miR-223 expression. Future studies are still required to decipher the detailed molecular mechanisms of KLF3-AS1 in gastric cancer.

## Introduction

Gastric cancer is a common human malignancy, and is mainly originated from the mucosa of the gastrointestinal tract (1–3). Gastric cancer is one of the most common malignant tumors with more than 1 million annual new cases and 0.8 million related deaths worldwide. Most patients with gastric cancer were diagnosed at advanced stages, which lead to poor prognosis, due to the non-specific symptoms of gastric cancer at the early stage (1–3). Chemotherapy and surgical resection were the main treatment regimens for the gastric cancer. Unfortunately, the patients who underwent surgical resection often experienced tumor recurrence, which largely contributed to the poor overall survival of patients with gastric cancer. In the patients with chemotherapy, platinum drugs, paclitaxel, 5-fluorouracil and Adriamycin were the commonly used therapeutic drugs. However, multi-drug resistance of gastric cancer cells often occurred in the patients, which may result in a poor prognosis (1–3). Up to date, the detailed mechanisms of gastric cancer progression remain inconclusive, which still requires further examination.

Long non-coding RNAs (lncRNAs) are RNA transcripts without protein coding capacity and are more than 200 nucleotides in length (4–6). LncRNAs participate various cellular functions including cell proliferation, invasion, migration, apoptosis and etc. LncRNAs have been shown to act as miRNA sponges competing with mRNA for miRNA binding (7). In the cancer biology, lncRNAs can act as tumor suppressors or oncogenes in the cancer development, and can serve as potential biomarkers for the cancer prognosis and diagnosis (4–6). In the gastric cancer, a large number of deregulated lncRNAs have been found to regulate gastric cancer progression, metastasis and chemo-resistance. Huang et al., found that lncRNA AK023391 promoted tumorigenesis and invasion of gastric cancer through activation of the PI3K/Akt signaling pathway (8); mesenchymal stem cell (MSC)-regulated lncRNAMACC1-AS1 promotes stemness and chemoresistance through fatty acid oxidation in gastric cancer (9). Wu et al., demonstrated that MSC-induced lncRNA HCP5 drove fatty acid oxidation through miR-3619-5p/AMPK/PGC1α/CEBPB axis to promote stemness and chemo-resistance of gastric cancer (10). Huang et al., revealed a positive feedback loop of lncRNA- PVT1 and FOXM1 that facilitated gastric cancer growth and invasion (11). Recently, the lncRNA KLF3-AS1 was identified as a tumor suppressor and suppressed cell migration and invasion in ESCC by impairing miR-185-5p-targeted KLF3 inhibition (12). However, the role of KLF3-AS1 in the gastric cancer has not been examined yet.

In this study, we aimed to determine the functional role of KLF3-AS1 in the gastric cancer cell proliferation, invasion, migration and chemosensitivity. In the experimental set up, various functional assays were employed to elucidate the mechanistic actions of KLF3-AS1 in gastric cancer progression. The present may shed some light on understanding into regulatory role of KLF3-AS1 in gastric progression.

## Materials and Methods

### Cell Culture

The normal gastric epithelial cell line (GES-1) and gastric cancer cell lines (SGC-7901, MNK-28 cells) were purchased from Cell Bank of Type Culture Collection of China (Shanghai, China). Human cisplatin-resistant gastric cancer cell line SGC7901/DDP cells were purchased from Shanghai Bogoo Biotechnology. Co., Ltd. (Shanghai, China). GES-1, SGC7901, MKN-28 and SGC-7901/DDP cells were cultured in RPMI-1640 medium (Thermo Fisher Scientific, Waltham, USA) which contained 10% fetal bovine serum (FBS; Thermo Fisher Scientific), penicillin (100 unit/ml) and streptomycin (100 μg/ml). All the cells were maintained in a humidified incubator containing 5% CO_2_ at 37°C.

### Plasmids, miRNAs, Cell Transfections, and Cisplatin Treatment

The KLF3-AS1-overexpressing plasmid (pcDNA3.1-KLF3-AS1) and pcDNA3.1-control were obtained from RiboBio (Guangzhou, China). The miRNAs including miR-223 mimics, miR-223 mimics control (mimics NC) were obtained from RiboBio (Guangzhou, China). Cell transfections were performed using the Lipofectamine 2000 reagent (Invitrogen, Carlsbad, USA) according to the manufacturer’s protocol. Cisplatin was purchased from Sigma-Aldrich (St. Louis, USA), and cisplatin at 1-1000 µM was used to treat gastric cancer cells for 24 h.

### Quantitative Real-Time PCR

Total RNA from cells and tumor tissues were extracted using TRIzol reagent (Invitrogen). A total of 1 µg RNA was reversely transcribed into cDNA by using the Primescript RT kit (Takara, Dalian, China). The real-time PCR was performed using a LightCycler^®^96 real-time PCR system linked to SYBR Premix EX Taq II kit (Takara) according to the manufacture’s protocol. The U6 and GAPDH were used as the respective reference controls for miRNA and mRNA expression. The relative expression levels were calculated using comparative Ct method.

### Cell Counting Kit-8 Assay

Cell viability of gastric cancer cells was determined by the Cell Counting Kit-8 (CCK-8) assay (Beyotime, Beijing, China). The gastric cancer cells including SGC-7901 and MKN-28 with different interventions for different time periods were incubated with CCK-8 solution at room temperature for 4 h. The cell viability of cells was determined by measuring optical density values at 450 nm.

### EdU Assay

Cell proliferative ability of gastric cancer cells was evaluated by 5′Ethynyl-2′-deoxyuridine (EdU) incorporation assay (Beyotime). The gastric cancer cells including SGC-7901 and MKN-28 with different interventions were seeded at 5 x 10^4^ cells/well, after 24 h culture, the cells were incubated with EdU solution for 2 h. After that, the cells were Apollo reaction cocktail and subsequently stained with 4′, 6-diamidino-2-phenylindole. The EdU-positive cells were evaluated using the fluorescent microscopy. The percentage of EdU-positive cells was calculated by dividing the number of EdU-positive cells by the number of DAPI-stained cells.

### Colony Formation Assay

Cell growth of gastric cancer cells was determined by the colony formation assay. Gastric cancer cells with different interventions were seeded in 6-well plates at a density of 1000 cells/well. The cells were growth on the plates for 10 days with RPMI 1640-10% FBS medium supplied. At the end of the experiment, the cells were stained with Giemsa for colony visualization. The number of colonies in each well was manually counted.

### Transwell Invasion Assay

Cell invasive ability of gastric cancer cells was performed by using Transwell invasion assay. Cells with different interventions in the serum-free medium were placed in the upper chamber (transwell inserts with 8-μm pore size polycarbonate coated with Matrigel), while the lower chamber was filled with RPMI-1640 medium supplied with 10% FBS. After a further incubation for 24 h, the cells invaded through the membrane were stained with 0.2% crystal violet. The number of invaded cells was manually counted using a light microscope.

### Wound Healing Assay

Cell migratory ability of gastric cancer cells was determined by wound healing assay. Gastric cancer cells with different interventions were plated on the 6-well plates and were cultured for monolayer formation followed by scratching with a sterile tip. After that, the cells were further culture for 48 h. The images of the cell monolayer were captured at 0 and 48 h after wound scratching. The wound healing was determined by (wound width at 0 h – wound width at 48 h)/wound width at 0 h x 100%.

### RNA Immunoprecipitation Assay

For pull-down assays of biotinylated miR-223, miR-223-mut and control were synthesized and transfected into SGC-7901 cells. SGC-7901 ells were lysed. According to manufacturer’s protocol, a portion of extract was used for input and another portion was incubated with Dynabeads M-280 Streptavidin (Invitrogen). RNA was purified with an RNA Isolation Kit (QIAGEN, Valencia, CA, USA) and used for qRT-PCR assays.

### Luciferase Reporter Assay

The binding sites between miR-223 and KLF3-AS1 were predicted by bioinformatics tools. Dual luciferase reporters were constructed by subcloning the targeted KLF3-AS1 including wild type and mutant segments into pGL3 reporter vector (Promega, Madison, USA). For the cell transfections, the SGC-7901 cells were co-transfected with respective miRNAs and reporter vectors. After 48 h, a Dual Luciferase Reporter Assay System (Promega) was applied to measure luciferase activity. Renilla luciferase activity was used for normalization.

### Xenograft Tumor Growth in the Nude Mice

The nude mice were purchased from Cyagen Co. Ltd., (Suzhou, China). The nude mice were 5–6-week-old females, weighing between 18 and 20 g, and were fed under specific pathogen-free conditions. The SGC-7901 cells transfected with pcDNA3.1 or pcDNA3.1-KLF3-AS1 were resuspended in serum-free RPMI-1640 medium at a concentration of 5 × 10^6^ cells/mL. Twelve 4-5-week-old male nude mice were randomly assigned to two groups, and each mouse was inoculated with 0.1 mL of cell suspension in the right axillary subcutis. The length and width of the tumor was measured weekly using a vernier caliper, and the tumor size was calculated as volume (mm3) = 0.5 × length (mm) × width^2^ (mm^2^). The mice were euthanized 5 weeks later, the tumors were collected and weighed, and the growth curve was calculated. The experimental process was under the supervision of the Ethics Committee of the First Affiliated Hospital of Wannan Medical College, and in accordance with internationally recognized guidelines on animal welfare.

### Expression and Survival Analysis of KLF3-AS1 in Gastric Cancer Patients

KLF3-AS1 expression in the gastric cancer tissues and the association between KLF3-AS1 expression and overall survival of the gastric cancer patients were analyzed using GEPIA (http://gepia.cancer-pku.cn/) based on TCGA RNA-Seq data (13).

### Statistical Analysis

The data were shown as mean ± standard deviation. Data were analyzed using GraphPad Prism version 8.0 software (GraphPad Software, La Jolla, USA). All the experiments were independently repeated for at least 3 times. Determination of the significance of differences was carried out using Student’s t-test or one-way analysis of variance followed by Bonferroni’s test. A P-value <0.05 was considered statistically significant.

## Results

### KLF3-AS1 Overexpression Suppressed Gastric Cancer Cell Viability and Proliferation

**Figure 1:**
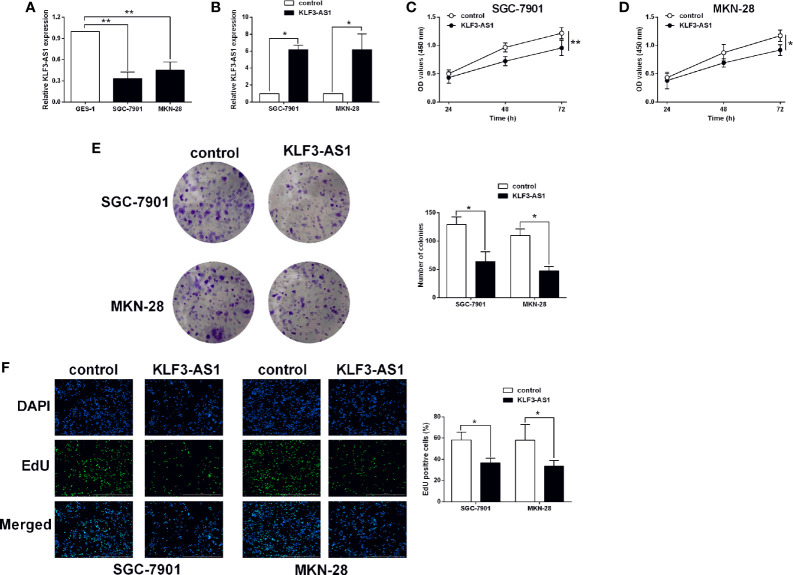
KLF3-AS1 overexpression suppressed gastric cancer cell viability and proliferation. **(A)** The expression levels of KLF3-AS1 in the GES-1, SGC-7901 and MKN-28 cells were examined by qRT-PCR (one-way ANOVA followed by Bonferroni’s test). **(B–F)** The gastric cancer cells including SGC-7901 and MKN-28 were transfected with pcDNA3.1 (control group) or pcDNA3.1-KLF3-AS1 (KLF3-AS1), and at 24 h after transfection, **(B)** KLF3-AS1 expression levels in the gastric cancer cells were determined by qRT-PCR; **(C, D)** cell viability of SGC-7901 and MKN-28 cells was determined by CCK-8 assay; **(E)** cell growth of SGC-7901 and MKN-28 cells was determined by colony formation assay; **(F)** cell proliferation of SGC-7901 and MKN-28 cells was determined by EdU assay (Student’s t-test). N = 3; *P < 0.05 and **P < 0.01.

The expression of KLF3-AS1 in GES-1, SGC-7901 and MKN-28 cells was examined by qRT-PCR, and KLF3-AS1 expression in SGC-7901 and MKN-28 cells was markedly lower than that in the gastric epithelial cell line, GES-1 (**Figure 1A**). Based on the publica data, KFL3-AS1 was exhibited a down-regulated trend in the gastric cancer tissues (**Supplemental Figure S1A**); while the expression of KFL3-AS1 was not correlated with the overall survival of patients with gastric cancer (**Supplemental Figure S1B**). The effects of KLF3 overexpression on the *in vitro* functions of gastric cancer cells were further determined. As shown in **Figure 1B**, pcDNA3.1-KLF3-AS1 transfection increased the KLF3-AS1 in the gastric cancer cells (SGC-7901 and MKN-28 cells) when compared to the pcDNA3.1 group. The CCK-8 assay results showed that KLF3-AS1 overexpression suppressed SGC-7901 and MKN-28 cell viability (**Figures 1C, D**). The cell growth of SGC-7901 and MKN-28 cells was also significantly attenuated by KLF3-AS1 overexpression as determined by colony formation assay (**Figure 1E**). The EdU assay showed that KLF3-AS1 overexpression reduced the cell proliferative ability of SGC-7901 and MKN-28 cells (**Figure 1F**).

### KLF3-AS1 Overexpression Suppressed Gastric Cancer Cell Invasion, Migration and EMT, but Promoted Chemosensitivity to Cisplatin

**Figure 2:**
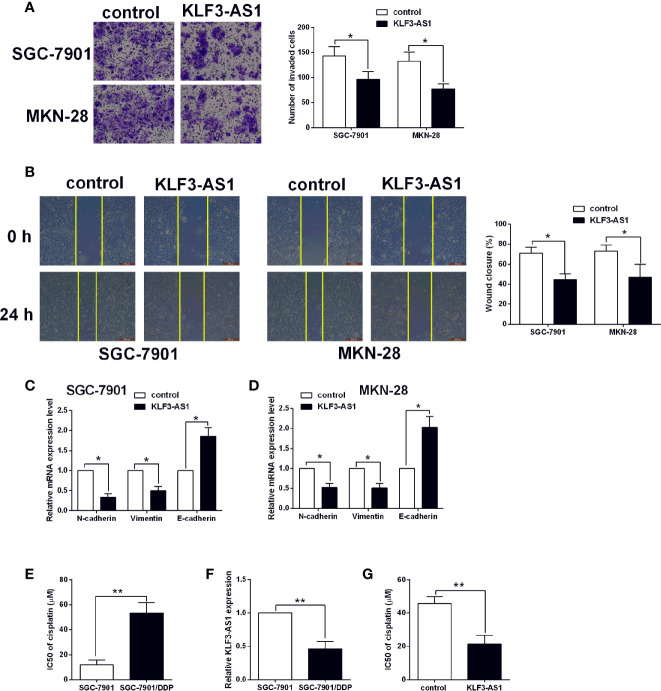
KLF3-AS1 overexpression suppressed gastric cancer cell invasion, migration and EMT, but promoted chemosensitivity to cisplatin. The gastric cancer cells including SGC-7901 and MKN-28 were transfected with pcDNA3.1 (control group) or pcDNA3.1-KLF3-AS1 (KLF3-AS1), and at 24 h after transfection, **(A)** cell invasion of SGC-7901 and MKN-28 cells was determined by Transwell invasion assay; **(B)** cell migration of SGC-7901 and MKN-28 cells was determined by wound healing assay; **(C, D)** the mRNA expression levels of N-cadherin, vimentin and E-cadherin in SGC-7901 and MKN-28 cells were determined by qRT-PCR; **(E)** The IC50 values of cisplatin in SGC-7901 and SGC-7901/DDP cells were determined by CCK-8 assay. **(F)** KLF3-AS1 expression level in SGC-7901 and SGC-7901/DDP cells was determined by qRT-PCR. **(G)** The IC50 values of cisplatin SGC-7901/DDP cells transfected with pcDNA3.1 or pcDNA3.1-KLF3-AS1 were determined by CCK-8 assay (Student’s t-test). N = 3; *P < 0.05 and **P < 0.01.

The transwell invasion assay showed that pcDNA3.1-KLF3-AS1 transfection in significantly reduced the number of invaded SGC-7901 and MKN-28 cells when compared to pcDNA3.1-transfected ones (**Figure 2A**). Consistently, the wound healing assay showed that KLF3-AS1 overexpression markedly attenuated the wound closure in both SGC-7901 and MKN-28 cells when compared to the control group (**Figure 2B**). The EMT-related markers including N-cadherin, vimentin, E-cadherin in SGC-7901 and MKN-28 cells were further determined by qRT-PCR. As shown in **Figures 2C, D**, KLF3-AS1 overexpression down-regulated the mRNA expression of N-cadherin and vimentin, but up-regulated E-cadherin in both SGC-7901 and MKN-28 cells. In terms of the chemosensitivity to cisplatin, the IC50 values of cisplatin in the SGC-7901/DDP cells were significantly higher than that in the SGC-7901 cells (**Figure 2E**). Moreover, the KLF3-AS1 expression in SGC-7901/DDP cells was up-regulated when compared to the SGC-7901 cells (**Figure 2F**). Furthermore, the CCK-8 assay revealed that KLF3-AS1 overexpression remarkably reduced the IC50 values of cisplatin in the SGC-7901/DDP cells when compared to control group (**Figure 2G**).

### KLF3-AS1 Repressed miR-223 Expression in Gastric Cancer Cells

**Figure 3:**
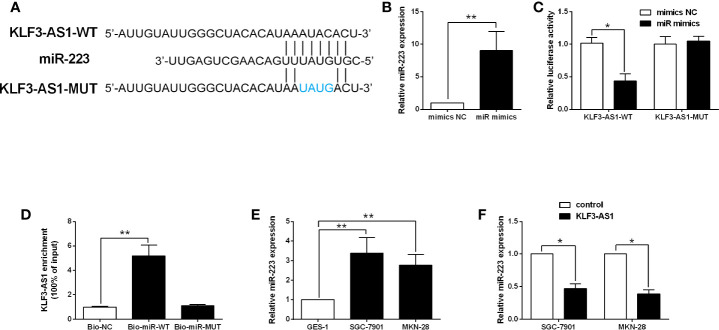
KLF3-AS1 repressed miR-223 expression in gastric cancer cells. **(A)** The predicted binding seed sequences between KLF3-AS1 and miR-223 were illustrated. **(B)** SGC-7901 cells were transfected with miR-223 mimics or mimics NC, and miR-223 expression level in SGC-7901 cells was determined by qRT-PCR at 24 h after transfection (Student’s t-test). **(C)** The luciferase reporter activity of KLF3-AS1-WT and KLF3-AS1-MUT in SGC-7901 cells with miR-223 mimics or mimics NC transfection was determined by Dual-Luciferase Report Assay kit (Student’s t-test). **(D)** The RIP determined the physical interaction between miR-223 and KLF3-AS1 (one-way ANOVA followed by Bonferroni’s test). **(E)** The expression levels of miR-223 in the GES-1, SGC-7901 and MKN-28 cells were examined by qRT-PCR (one-way ANOVA followed by Bonferroni’s test). **(F)** The gastric cancer cells including SGC-7901 and MKN-28 were transfected with pcDNA3.1 (control group) or pcDNA3.1-KLF3-AS1 (KLF3-AS1), and at 24 h after transfection, the miR-223 expression level was determined by qRT-PCR (Student’s t-test). N = 3; *P < 0.05 and **P < 0.01.

The targets of KLF3-AS1 were further examined by bioinformatics prediction (StarBase V2.0) (14), and miR-223 was chosen for further examination as its effect on the gastric cancer progression has been demonstrated in previous studies (15, 16). The binding seed sequences between miR-223 and KLF3-AS1 were shown in **Figure 3A**. Further luciferase reporter assay revealed that miR-223 overexpression repressed the luciferase activity of KLF3-AS1-WT vector, but had no effect on the luciferase activity of KLF3-AS1-MUT vector (**Figures 3B, C**). The RIP further confirmed the interaction between miR-223 and KLF3-AS1 (**Figure 3D**). The qRT-PCR examined the expression of miR-223 in GES-1, SGC-7901 and MKN-28 cells, and miR-223 expression levels in the SGC-7901 and MKN-28 cells were significantly higher than that in the GES-1 cells (**Figure 3E**). In addition, KLF3-AS1 overexpression caused a significantly decrease in the miR-223 expression levels of SGC-7901 and MKN-28 cells (**Figure 3F**).

### MiR-223 Attenuated the Effects of KLF3-AS1 on the Gastric Cell Viability, Proliferation

**Figure 4:**
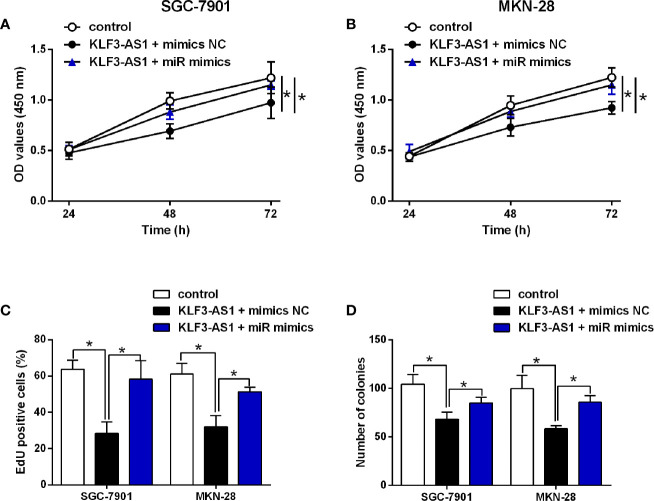
MiR-223 attenuated the effects of KLF3-AS1 on the gastric cell viability, proliferation. SGC-7901 and MKN-28 cells were co-transfected with pcDNA3.1 + mimics NC (control group), pcDNA3.1-KLF3-AS1 + mimics NC, or pcDNA3.1-KLF3-AS1 + miR-223 mimics, at 24 h after transfection, **(A, B)** cell viability of SGC-7901 and MKN-28 cells was determined by CCK-8 assay; **(C)** cell proliferation of SGC-7901 and MKN-28 cells was determined by EdU assay; **(D)** cell growth of SGC-7901 and MKN-28 cells was determined by colony formation assay (one-way ANOVA followed by Bonferroni’s test). N = 3; *P < 0.05.

The *in vitro* rescue studies were carried to determine if miR-223 could impact on the KLF3-AS1-mediated actions in the gastric cancer cellular functions. The CCK-8 assay showed that KLF3-AS1 overexpression inhibited the cell viability of SGC-7901 and MKN-28 cells, which was significantly attenuated by miR-223 mimics transfection (**Figures 4A, B**). MiR-223 overexpression significantly attenuated the inhibitory effects of KLF3-AS1 overexpression on the cell proliferation and growth of SGC-7901 and MKN-28 cells (**Figures 4C, D**).

### MiR-223 Attenuated the Effects of KLF3-AS1 on the Gastric Cell Invasion, Migration, EMT and Chemosensitivity

**Figure 5:**
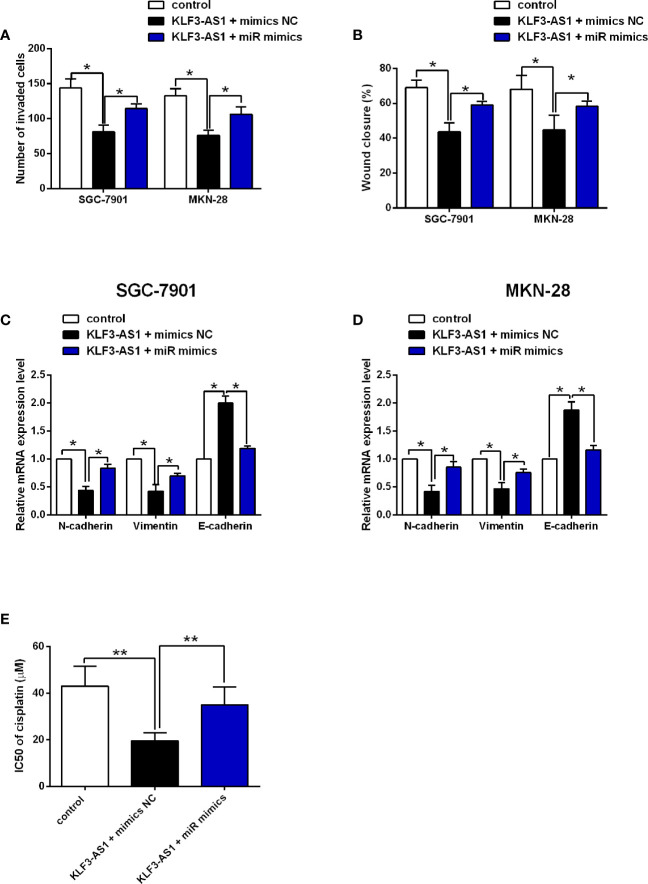
MiR-223 attenuated the effects of KLF3-AS1 on the gastric cell invasion, migration, EMT and chemosensitivity. SGC-7901 and MKN-28 cells were co-transfected with pcDNA3.1 + mimics NC (control group), pcDNA3.1-KLF3-AS1 + mimics NC, or pcDNA3.1-KLF3-AS1 + miR-223 mimics, at 24 h after transfection, **(A)** cell invasion of SGC-7901 and MKN-28 cells was determined by Transwell invasion assay; **(B)** cell migration of SGC-7901 and MKN-28 cells was determined by wound healing assay; **(C, D)** the mRNA expression levels of N-cadherin, vimentin and E-cadherin in SGC-7901 and MKN-28 cells were determined by qRT-PCR. **(E)** SGC-7901/DDP cells were co-transfected with pcDNA3.1 + mimics NC (control group), pcDNA3.1-KLF3-AS1 + mimics NC, or pcDNA3.1-KLF3-AS1 + miR-223 mimics, the IC50 values of cisplatin in SGC-7901/DDP cells were determined by CCK-8 assay (one-way ANOVA followed by Bonferroni’s test). N = 3; *P < 0.05 and **P < 0.01.

The transwell invasion and wound healing assay results revealed that KLF3-AS1 overexpression repressed the invasion and migration of SGC-7901 and MKN-28 cells, which was markedly reversed by miR-223 overexpression (**Figures 5A, B**). Consistently, KLF3-AS1 overexpression down-regulate the mRNA expression of N-cadherin and vimentin, but up-regulated the mRNA expression of E-cadherin, which was attenuated by miR-223 overexpression in both SGC-7901 and MKN-28 cells (**Figures 5C, D**). The effects of KLF3-AS1/miR-223 signaling on the chemosensitivity to cisplatin were further examined in SGC-7901/DDP cells. As shown in **Figure 5E**, KLF3-AS1 overexpression reduced the IC50 of cisplatin in SGC-7901/DDP cells, which was significantly increased by miR-223 overexpression (**Figure 5E**).

### KLF3-AS1 Overexpression Suppressed *In Vivo* Tumor Growth of SGC-7901 Cells

**Figure 6:**
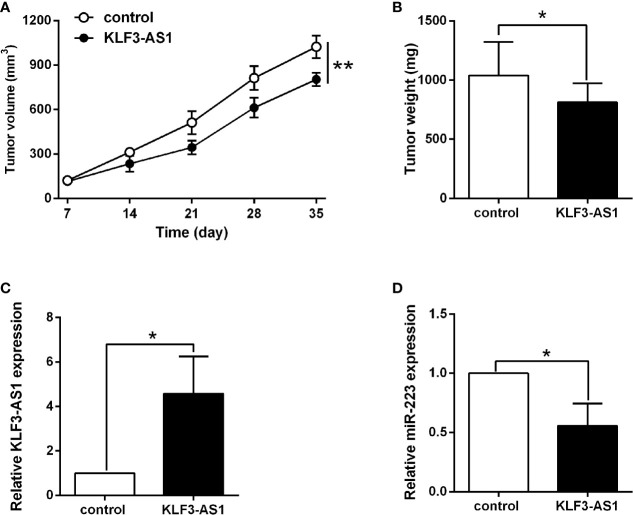
KLF3-AS1 overexpression suppressed *in vivo* tumor growth of SGC-7901 cells. **(A)** The *in vivo* tumor growth of SGC-7901 cells transfected with pcDNA3.1 or pcDNA3.1-KLF3-AS1 was examined in the xenograft nude mice model. **(B)** The tumor weight of the dissected tumors was examined at the end of the experiment. **(C)** KLF3-AS1 and **(D)** miR-223 expression in dissected tumor tissues were examined by qRT-PCR (Student’s t-test). N = 6; *P < 0.05 and **P < 0.01.

The effects of KLF3-AS1 on the gastric cancer progression were further examined in the xenograft nude mice model. As shown in **Figure 6A**, KLF3-AS1 overexpression significantly attenuated the tumor growth of SGC-7901 cells in the nude mice when compared to the control group, and consistent findings were also detected in the tumor weight (**Figure 6B**). Moreover, the KLF3-AS1 and miR-223 expression levels in the dissected tumor tissues were examined by qRT-PCR, and the results showed that KLF3-AS1 was significantly up-regulated, and miR-223 was significantly down-regulated in the pcDNA3.1-KLF3-AS1 group when compared to the pcDNA3.1 group (**Figures 6C, D**).

## Discussion

Gastric cancer is a deadly disease, and the low rate of early diagnosis and chemoresistance largely contributed to the poor prognosis of gastric cancer (17, 18). LncRNAs have been extensively reported for their roles in regulating cancer progression (19). In this study, we found that KLF3-AS1 was down-regulated in gastric cancer cells. Overexpression of KLF3-AS1 repressed gastric cancer cell proliferation, growth. In addition, KLF3-AS1 overexpression also exerted inhibitory effects on the gastric cancer cell invasion, migration and EMT, but promoted chemosensitivity of gastric cancer cells to cisplatin. The mechanistic studies showed that KLF3-AS1 could act as the “sponge” for miR-223 and to repress miR-223 expression in gastric cancer cells. Overexpression of miR-223 reversed the inhibitory effects of KLF3-AS1 overexpression on gastric cancer cell proliferation, invasion, migration and EMT, and attenuated the enhanced effects of KLF3-AS1 overexpression on chemosensitivity to cisplatin. The *in vivo* studies showed that KLF3-AS1 overexpression suppressed the tumor growth of SGC-7901 in the nude mice. Taken together, the present suggested the tumor-suppressive role of KLF3-AS1 in gastric cancer, where KLF3-AS1 exerted its effects *via* targeting miR-223.

To our best knowledge, the role of lncRNAs in regulating gastric cancer has been reported in a large number of studies. The lncRNAs including PCAT-1, DANCR, SNHG5, GEHT1, ANRIL and so on were identified as oncogenes to promote the gastric cancer cell progression and chemoresistance (20–24); on the other hand, the lncRNAs such as CASC2 and CRAL have been found to act as a tumor-suppressor to attenuate gastric cancer progression (25). In our study, we found that KLF3-AS1 was down-regulated in the gastric cancer cells, and our functional studies further suggested that KLF3-AS1 could act as a tumor suppressor in the gastric cancer cells. Based on the previous literatures, several studies have determined the biological functions of KLF3-AS1 in different types of diseases. Liu et al., showed that exosomal KLF3-AS1 from MSCs promoted cartilage repair and chondrocyte proliferation in osteoarthritis (26). Mao et al., showed that KLF3-AS1 in human mesenchymal stem cell-derived exosomes ameliorated pyroptosis of cardiomyocytes and myocardial infarction through miR-138-5p/Sirt1 axis (27). Recently, studies found that KLF3-AS1 acted as a tumor suppressor to inhibit the cell migration and invasion in ESCC, which was in consistent with our findings.

The “miRNA sponge” actions for lncRNAs have been well-documented in various reports. In terms of KLF3-AS1, KLF3-AS1 could act as an endogenous RNA for miR-138-5p, miR-206, miR-185-5p, respectively (12, 26–29). In our study, we further found that KLF3-AS1 could act as the “sponge” for miR-223 and to repress miR-223 expression in gastric cancer cells. In the studies of miR-223, Li et al., showed that miR-223 could function as an oncogene in human gastric cancer by targeting FBXW7/hCdc4 (30). Liu also showed that miR-223-5p targeted long non-coding RNA TP73 antisense RNA1 to promote the invasion of gastric cancer (30). In terms of chemo-resistance, miR-223 could promotes the cisplatin resistance of human gastric cancer cells *via* regulating cell cycle by targeting FBXW7 (31). In addition, exosomal transfer of macrophage-derived miR-223 promoted doxorubicin resistance in gastric cancer (32). In our results, we showed that miR-223 overexpression could attenuate the tumor-suppressive actions of KLF3-AS1 in the gastric cancer cells, and also reversed the enhanced effects of KLF3-AS1 on the chemosensitivity to cisplatin in gastric cancer cells. These results may imply that KLF3-AS1 acted as an endogenous RNA for miR-223 to regulate the gastric cancer progression and chemoresistance. Nevertheless, the *in vivo* studies only determined the effects of KFL3-AS1 overexpression on the tumor growth of the nude mice, and future studies should examine if KFL3-AS1 overexpression could attenuate the *in vivo* metastasis of gastric cancer cells in the nude mice, and the *in vivo* mechanistic studies should be considered in the future plans. As miRNA could target various downstream targets by interacting with the 3’ untranslated region of the respective mRNAs (**Supplemental Table 1**), future studies should perform mechanistic studies to further reveal the regulatory role of KFL3-AS1/miR-223 in gastric cancer progression. In addition, the present study only used two gastric cancer cell lines, and future studies should include more gastric cancer cell lines to confirm current findings.

In conclusion, our results for the first time demonstrated that KLF3-AS1 was down-regulated in gastric cancer cells and repressed gastric cancer cell proliferation, invasion, migration and EMT, and enhanced chemosensitivity to cisplatin. Further mechanistic results indicated that KLF3-AS1 exerted its biological function in gastric cancer cells by inhibiting miR-223 expression. Future studies are still required to decipher the detailed molecular mechanisms of KLF3-AS1 in gastric cancer.

## Data Availability Statement

The original contributions presented in the study are included in the article/**Supplementary Material**. Further inquiries can be directed to the corresponding author.

## Ethics Statement

The experimental process was under the supervision of the Ethics Committee of the First Affiliated Hospital of Wannan Medical College.

## Author Contributions

HJ and LL designed the study and supervised the whole project. HJ, KH, and YX performed the experiments and analyzed the data. LL and XZ drafted the manuscript. HJ revised the drafted manuscript. All authors contributed to the article and approved the submitted version.

## Funding

This study was supported by the 2020 Key Projects of Natural Science in Colleges and Universities of Anhui Province (KJ2020A0597).

## Conflict of Interest

The authors declare that the research was conducted in the absence of any commercial or financial relationships that could be construed as a potential conflict of interest.

## Publisher’s Note

All claims expressed in this article are solely those of the authors and do not necessarily represent those of their affiliated organizations, or those of the publisher, the editors and the reviewers. Any product that may be evaluated in this article, or claim that may be made by its manufacturer, is not guaranteed or endorsed by the publisher.

## Supplementary Material

Supplementary Figure 1 Expression and survival analysis of KLF3-AS1 in gastric cancer. **(A)** The expression of KLF3-AS1 in gastric cancer tissues and normal gastric tissues. **(B)** The association between KLF3-AS1 expression and overall survival of gastric cancer patients was indicated by the Kaplan-Meier analysis.
