# Supplementary figures and images for: Long Noncoding RNA KLF3-AS1 Acts as an Endogenous RNA of miR-223 to Attenuate Gastric Cancer Progression and Chemoresistance
Source: Front Oncol. 2021 Oct 21;11:704339. doi: 10.3389/fonc.2021.704339 (PMC8567101; doi:10.3389/fonc.2021.704339)

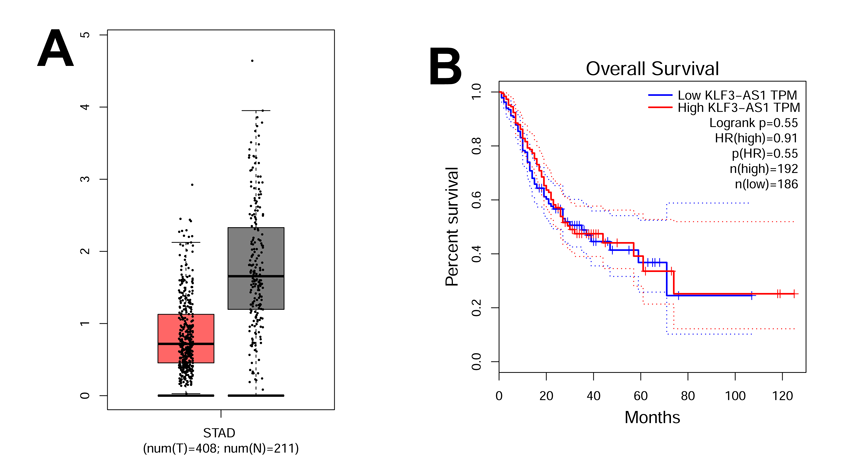

Supplement: Supplementary Figure 1 — Expression and survival analysis of KLF3-AS1 in gastric cancer. (A) The expression of KLF3-AS1 in gastric cancer tissues and normal gastric tissues. (B) The association between KLF3-AS1 expression and overall survival of gastric cancer patients was indicated by the Kaplan-Meier analysis. [file Image_1.tif]
